# miR-1275 Delivered via Mesenchymal Stem Cell-Derived Extracellular Vesicles Regulates ER-Phagy Through AXIN2 in Nucleus Pulposus Cells

**DOI:** 10.1155/sci/5091529

**Published:** 2025-05-29

**Authors:** Zhiwu Dong, Hailong Zhang, Wenwei Yang, Keliang Huang, Xin Zhang, Lianxiang Xing, Ying Zhang, Kewen Zhao

**Affiliations:** ^1^Department of Laboratory Medicine, Shanghai Second People's Hospital, Shanghai 200011, China; ^2^Department of Orthopedics, Shanghai Tenth People's Hospital, Tongji University School of Medicine, No. 301, Yanchang Road, Shanghai 200072, China; ^3^Department of Orthopedics, Putuo District People's Hospital, Shanghai 200060, China; ^4^Key Laboratory of Cell Differentiation and Apoptosis of National Ministry of Education, Shanghai Frontiers Science Center of Cellular Homeostasis and Human Diseases, Department of Pathophysiology, Shanghai Jiao Tong University School of Medicine, Shanghai 200025, China

**Keywords:** apoptosis, AXIN2, ER-phagy, ER-stress, intervertebral disc degeneration, miR-1275

## Abstract

Intervertebral disc degeneration (IDD) is a major contributor to low back pain, a prevalent and debilitating condition. Nucleus pulposus (NP) cells are essential for maintaining disc homeostasis, and their dysfunction plays a crucial role in IDD development. This study aimed to explore the potential role of miR-1275, delivered via mesenchymal stem cell-derived extracellular vesicles (MSCs-EVs), in IDD pathogenesis and to elucidate the underlying molecular mechanisms through *in vitro* investigations. Decreased miR-1275 expression and elevated endoplasmic reticulum (ER) stress were observed in degenerated human NP tissues compared to normal controls. An *in vitro* IDD model was established by treating NP cells (NPCs) with advanced glycation end products (AGEs). Subsequent experiments demonstrated that EVs from miR-1275-overexpressing MSCs reduced AGE-induced ER stress, extracellular matrix (ECM) degradation, and apoptosis in NPCs by enhancing ER-phagy. Bioinformatic analyses identified AXIN2 as a direct target of miR-1275. Remarkably, AXIN2 overexpression significantly attenuated the effects of miR-1275 on NPC proliferation, apoptosis, ER stress, and ER-phagy under AGE-induced conditions. Mechanistic studies validated AXIN2 as a target of miR-1275, with miR-1275 binding to the 3′ untranslated region of AXIN2 and regulating its expression. Collectively, our *in vitro* findings reveal that MSCs-EVs carrying miR-1275 can modulate ER stress and enhance ER-phagy in NPCs through the targeted downregulation of AXIN2, suggesting a potential molecular mechanism in IDD pathogenesis.

## 1. Introduction

Intervertebral disc degeneration (IDD) is a prevalent chronic spinal disorder, leading to several clinical manifestations, including cervical spondylosis, intervertebral disc herniation, and low back pain [1, 2]. IDD severely impacts the quality of life and poses a significant burden on healthcare systems worldwide [3]. It is characterized by the progressive loss of disc space and endplate sclerosis [4]. Multiple factors contribute to the pathogenesis of IDD, such as genetic predisposition, biochemical and environmental factors, inflammation, and lifestyle factors such as aging, mechanical overload, obesity, and smoking.

The nucleus pulposus (NP) constitutes the central part of the intervertebral disc, and the NP cells (NPCs) play a crucial role in maintaining the homeostasis of extracellular matrix (ECM) proteins [5]. Preserving an adequate number of functional NPCs is essential for normal intervertebral disc functioning. Therefore, a reduced number of NPCs and impaired functioning of the remaining NPCs may lead to a degenerated disc [6]. Moreover, oxidative stress in NPCs can result in endoplasmic reticulum (ER) stress, which may subsequently lead to excessive apoptosis and senescence through the production of reactive oxygen species (ROS). Alternatively, advanced glycation end products (AGEs) accumulate in degenerative tissues, and increased levels of AGEs are associated with ER stress. Collectively, these damaging processes contribute to the development of IDD [7–9]. ER-phagy, the autophagic process that selectively incorporates components of the ER into autophagosomes, is involved in ER homeostasis. In general, autophagy regulates homeostasis under various conditions and can exert either pro-survival or pro-death effects depending on the specific context. In a rat model of neuropathic pain, ER stress-induced ER-phagy impairment was observed in the spinal cords of these rats. Targeting ER-phagy through pharmacological intervention yielded beneficial effects in this model [10].

Current treatment options for IDD, such as conservative management and surgical intervention, may provide symptomatic relief, but they fail to address the underlying pathophysiological mechanisms driving the degenerative process. Consequently, there exists a significant unmet clinical need for novel therapeutic strategies targeting IDD.

Mesenchymal stem cells (MSCs) have demonstrated potential therapeutic effects in IDD, as studies have shown their ability to inhibit apoptosis in NPCs [11]. MSCs secrete extracellular vesicles (EVs), which are membrane-bound structures derived from cells, comprising exosomes and microvesicles. These EVs originate primarily from the endosomal system and carry diverse molecular cargoes, including microRNAs (miRNAs), mRNAs, and proteins, from parent cells to target cells, thereby facilitating intercellular communication. Notably, EVs derived from MSCs have been demonstrated to inhibit apoptosis in NPCs [12, 13], although the precise underlying mechanism remains unknown.

MiRNAs, a class of endogenous noncoding RNAs, are crucial regulators of numerous cellular processes, such as proliferation and apoptosis. They exert their regulatory effects by binding to target mRNAs, thereby modulating gene expression. Within the context of the intervertebral disc, miRNAs have been shown to regulate NPCs proliferation, extracellular matrix synthesis, and apoptosis [14], indicating their pivotal role in the development of skeletal pathophysiology. Furthermore, *in vivo* studies have corroborated the dysregulation of distinct miRNAs in patients with IDD, with some miRNAs being upregulated while others are downregulated, strongly suggesting that miRNAs play a significant role in the etiopathogenesis of IDD [15–18].

The aim of this study was to identify the miRNA that modulates AGEs-induced apoptosis and ER stress in NPCs as a model of IDD and to investigate the potential regulatory mechanisms by which this miRNA exerts its effects during this process.

## 2. Materials and Methods

### 2.1. Specimen Selection

Human NP tissues were obtained from patients who underwent spinal surgery at Putuo District People's Hospital from March 2021 to October 2022. Written informed consent was obtained from all participants or their legal guardians prior to sample collection, and the study was conducted in accordance with the Declaration of Helsinki. The degree of disc degeneration in the patients was determined by the Pfirrmann grading system using preoperative MRI images. NP tissues from nine patients (grade I-II, 6 males and 3 females, aged 10–25 years) with acute lumbar disc herniation or adolescent idiopathic scoliosis but without IDD were considered relatively normal, whereas degenerated NP tissue was obtained from 25 patients (grade III–V, 14 males and 11 females, aged 26–78 years) with disc degeneration. All experimental protocols were approved by the Research Ethics Committee of Putuo District People's Hospital (ethical number: 2023 Trial No. 003).

### 2.2. Cell Isolation and Culture

Healthy human NPCs from the human intervertebral disc were purchased from Sciencell Research Laboratories (Cat# 4800, Carlsbad, CA, USA), and cultivated in DMEM (Gibco, Grand Island, NY, USA) containing 10% heated-inactivated fetal bovine serum (FBS, Gibco), 1% endothelial cell growth factor (Beyotime, China), 100 IU/mL penicillin and streptomycin (Sigma) at 37°C in a humidified atmosphere of 5% CO_2_.

### 2.3. Isolation and Identification of Mesenchymal Stem Cells

Bone marrow cells from healthy subjects—who provided informed consent for the use of their specimens—were collected and diluted in an equal volume of PBS. Isolation of MSCs was carried out by density gradient centrifugation in 1.077 g/mL Ficoll (Sigma–Aldrich Chemical Company) at 2000 rpm for 20 min and by adherence to tissue culture plastic. Cells were cultured in DMEM with 10% extracellular vesicle-free fetal bovine serum and 1% penicillin-streptomycin in a 5% CO_2_ incubator at 37°C. DMEM was renewed on the 3rd day of cell culture and every 2 days thereafter. Cells at passages 2, 3, or 4 were used for experiments. MSCs were identified by the positive expression of the cell surface markers CD73, CD90, and CD105 and the lack of expression of CD45, CD34, and CD11b using flow cytometry (BD FACSLyric; Becton Dickinson Biosciences, San Jose, CA). The potential to differentiate into osteocytes, chondrocytes, and adipocytes was determined by culturing MSCs in different media (Cyagen, China). Subsequently, Alizarin red staining, Oil red O staining, and Alcian blue staining were performed to confirm the differentiation of each lineage.

### 2.4. Isolation and Identification of MSCs-Derived EVs

At a confluence of ~80% of MSCs, the culture medium was replaced with DMEM. After 48 h, the conditioned medium was collected and subjected to differential centrifugation steps. Briefly, the medium was first centrifuged at 500 g for 10 min at 4°C to remove cells, followed by 12,000 g for 20 min to remove cell debris and larger vesicles. The supernatant was then filtered through a 0.22 µm membrane and ultracentrifuged at 100,000 g for 2 h at 4°C to collect EVs. The EV pellet was washed once with PBS and resuspended in sterile PBS. The isolated EVs were characterized using multiple complementary approaches. Morphological characterization was performed using transmission electron microscope (TEM). Size distribution and particle concentration were analyzed using nanoparticle tracking analysis (NTA) with NanoSight NS-300 (NanoSight Technology, Malvern, UK). The presence of characteristic EV-associated proteins including CD63, CD9, CD81, and TSG101 was confirmed by Western blotting analysis.

### 2.5. Internalization of Dil-Labeled EVs into NPCs

EVs were labeled with red fluorescent dye (Dil; Sigma–Aldrich Chemical Company) according to the manufacturer's protocol. Dil-labeled EVs were incubated with NPCs at 37°C for 24 h. After 24 h, NPCs were washed twice with PBS and subsequently fixed with 4% paraformaldehyde at room temperature for 10 min, and the nuclei were stained with DAPI at room temperature for 15 min. Cells were analyzed by fluorescent microscopy (Nikon Corporation, Tokyo, Japan).

### 2.6. Cell Transfection and EVs Treatment

Plasmid constructs were purchased from GenePharma (Shanghai, China). MSCs were transfected with negative control (NC)-mimic or miR-1275-mimic using Lipofectamine 3000 (Invitrogen, Carlsbad, CA, USA). NPCs were incubated with EVs from transfected MSCs and stimulated with AGEs. NPCs incubated with EVs from untransfected MSCs were used as control.

### 2.7. Reverse Transcription-Quantitative Polymerase Chain Reaction (RT-qPCR)

Total RNA from NPCs was isolated using a TRIzol kit (Invitrogen, Carlsbad, CA, USA) according to the manufacturer's instructions. RNA was reverse transcribed into cDNA using Transcriptor First Strand cDNA Synthesis kit (TaKaRa Biotechnology, Dalian, China). Quantitative PCR was performed using TaqMan sequence, probe and SYBR Green II (TaKaRa, Tokyo, Japan) in an ABI PRISM 7900 Sequential Dectection System (Applied Biosystems, Foster City, CA, USA) with the following reaction steps: pre-denaturation at 95°C for 10 min and 40 cycles of denaturation at 95°C for 10 s, annealing at 60°C for 20 s and extension at 72°C for 34 s. The primer sequences are listed in Table 1. The 2^*−ΔΔCt*^ method was used to analyze the data.

### 2.8. Cell Couting Kit-8 (CCK-8) Assay

Cell counting kit-8 (CCK-8) reagent (Dojindo Molecular Technologies, Kumamoto, Japan) was added to NPCs in 96-well plates (1500 cells/well), and cells were incubated for 2 h at 37°C in the dark. The OD value at 450 nm was measured using a multiplate reader (Thermo Fisher, Waltham, MA, USA).

### 2.9. Flow Cytometry

NPCs (1 × 10^6^ cells/mL) were washed with PBS and incubated with the antibodies and the isotype control set for 30 min at room temperature in the dark. For analysis of apoptosis, cells were incubated with Annexin V-APC and propidium iodide (PI) of the Annexin V-APC apoptosis detection kit (BD Pharmingen, San Diego, USA). After incubation, cells were washed twice with PBS and fluoresecent intensity of the cells was measured by the FACSFlow Cytometer (BD, San Diego, CA, USA) with the isotype control as negative control.

### 2.10. Fluorescence In Situ Hybridization (FISH)

Exiqon QIAGEN (Hilden, Germany) was used to design and synthesize a digoxin-labeled miR-1275 probe with locked nucleic acid modifications. A FISH kit (Exiqo-QIAGEN) was used in NPCs and fluoresencent signals were detected by confocal microscopy (Zeiss LSM 710, Germany).

### 2.11. Dual Luciferase Reporter Assay

3′ UTR fragments of AXIN2 mRNA with wild-type or mutated binding sites for miR-1275 were inserted into a luceriferase reporter vector (Promega, Madison, MI, USA). NPCs (8 × 10^3^cells/well in a 96-well plate) were transfected with wild-type or mutated plasmids using Lipofectamine 3000. In addition, cells were transfected with miR-1275 expression plasmid or a control plasmid. After 48 h, a dual luciferase reporter gene analysis system (Promega, Madison, WI, USA) was used to measure luciferase activity. Opical density values were determined by a multi-plate reader. The relative luciferase activity was calculated as the ratio of firefly compared with renilla luciferase activity.

### 2.12. Immunofluorescence Analysis

NPCs were mounted on glas coverslips and fixed with 4% paraformaldehyde. Cells were permeabilized with 0.2% Triton X-100 in PBS, and slides were washed with PBS and blocked with 2% BSA in PBS at 37°C for 2 h. Cells were incubated with primary antibodies and after incubation, washed twice with PBS. Then, cells were incubated with secondary antibodies at 37°C for 2 h. Staining for nuclei was done for 5 min with DAPI. Images were taken using a confocal microscope (Zeiss LSM).

### 2.13. Western Blotting

NPCs or EVs were lysed in RIPA lysis buffer containing protease inhibitor cocktail (Sigma–Aldrich) on ice for 30 min. After centrifugation (10 min at 13,000 rpm), the supernatant was removed, and protein concentration was measured using the BCA kit (Pierce, Rockford, IL, USA). Proteins were then separated by SDS-PAGE and transferred onto PVDF membranes. The membranes were blocked in TBST with 5% milk for 1 h and subsequently incubated with the following primary antibodies against: GRP78 (Abcam, ab108615, 1:5000); CHOP (Abcam, ab11419, 1:1000), p-IRE1*α* (Abcam, ab124945, 1:1000); IRE1*α* (Abcam, ab96481, 1:1000); cleaved caspase-3 (Abcam, ab32042, 1:500); caspase-3 (Abcam, ab32351, 1:5000), Bcl-2 (Abcam, ab182858, 1:2000); Bax (Abcam, ab32503, 1:500); aggrecan (abcam, ab3778, 1:1000); collegan II (Abcam, ab34712, 1:1000); MMP13 (Abcam, ab39012, 1:3000); ADAMTS5 (Abcam, ab182795, 1:1000); CD9 (Abcam, ab92726, 1:2000); CD63 (Abcam, ab134045, 1:1000); CD81 (Abcam, ab79559, 1:1000); TSG101 (Abcam, ab125011, 1:500); calnexin (Abcam, 92573, 1:20,000); FAM134B (Abcam, ab246913, 1:1000); Beclin1 (Abcam, ab207612, 1:2000), P62 (Abcam, ab109012 1:10,000); LC3II/I (CST, #4108,1:1000); and GAPDH (Abcam, ab8245, 1:6000). After incubation at 4°C overnight, membranes were washed with TBST for three times, and then incubated with HRP-labeled secondary antibody for 1 h at room temperature. Finally, enhanced chemiluminescence kit (Thermo Scientific, Waltham, MA, USA) was used to visualize protein bands, which were quantified using Image J software (USA NIH, Bethesda, MD).

### 2.14. RNA Pull-down Assay

Biotin-labeled wild-type and mutated AXIN2 were transfected into NPCs. After 48 h, cells were washed with PBS and lysed in lysis buffer (20 mM Tris, pH 7.5, 200 mM NaCl, 2.5 mM MgCl_2_, 0.05% IGEPAL CA-630 and 1 mM dithiothreitol) containing protease inhibitor and ribonuclease inhibitor on ice for 10 min. The lysate was then incubated with magnetic beads coated with streptavidin (Invitrogen, Carlsbad, CA, USA) at 4°C overnight. Prior to incubation, magnetic beads were precoated with RNase free BSA and yeast tRNA (Sigma–Aldrich Chemical company, St Louis, MO, USA). Bound RNA was analyzed by qRT-PCR.

### 2.15. RNA Immunoprecipitation (RIP) Assay

NPCs were lysed with NP-40 lysis buffer consisting of 1 mMphenylmethylsulfonyl fluoride, 1 mM dithiothreitol, 1% protease inhibitor cocktail (Sigma–Aldrich Chemical company), and RNase inhibitor (Invitrogen) on ice for 5 min. One part of cell extraction solution served as an input and the other part was incubated with RIP buffer containing magnetic beads (EZ-Magna RIP kit; Millipore, MA, USA) conjugated with anti-Argonaute 2 (Abcam, Cambridge, UK) or immunoglobulin G (Millipore, Bedford, MA, USA). The bound proteins to the complex were lysed with proteinase K buffer, and bound RNA was isolated and analyzed by qRT-PCR.

### 2.16. ER-Tracker and Lyso-Tracker Staining

ER and lysosome colocalization in ER-phagy was assessed by fluorescent labeling of ER and lysosomes. NPCs were mounted on glass coverslips in a 6-well plate. After treatment, cells were stained with ER-tracker green (Yisheng Biotech, Shanghai, China), Lysotracker red (Yisheng), and DAPI at 37°C for 30 min. NPCs were washed three times with PBS and fluorescence was visualized by fluorescent microscopy.

### 2.17. Statistical Analysis

All experiments were performed in triplicate and data are presented as mean ± standard deviation (SD). Statistical analyses were performed using GraphPad Prism 8.0 software (GraphPad Software Inc., San Diego, CA, USA). For comparisons between two groups, unpaired Student's *t*-test was used. For multiple group comparisons, one-way ANOVA was performed followed by Tukey's post hoc test. The Benjamini–Hochberg procedure was applied to control the false discovery rate (FDR) in multiple comparisons. Results were considered statistically significant when adjusted *p*-values (padj) were < 0.05.

## 3. Results

### 3.1. Isolation and Characterization of Human Mesenchymal Stem Cells

**Figure 1:**
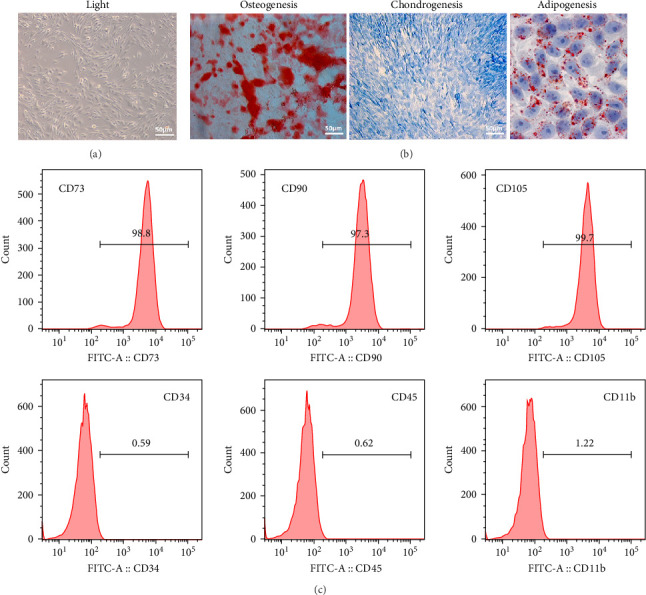
Isolation and characterization of hMSCs. (A) Morphology of MSCs by optical microscopy (scale bar: 50 μm). (B) hMSCs were induced to differentiate into osteoblasts, chondrocytes, and adipocytes (scale bar: 50 μm). (C) Positive markers (CD73, CD90, and CD105) and absence of hematopoietic markers (CD45, CD34, and CD11b) of MSCs were measured by flow cytometry. hMSCs, human mesenchymal stem cells.

Characterization of human MSCs (hMSCs) were isolated from bone marrow aspirates and their characteristic morphology was confirmed under optical microscopy (Figure 1A). The isolated hMSCs were cultured and subsequently induced to differentiate into osteoblasts, chondrocytes, and adipocytes, as evidenced by their respective lineage-specific staining patterns (Figure 1B). Flow cytometric analysis validated the expression of characteristic MSC surface markers (CD73, CD90, and CD105) and the absence of hematopoietic markers (CD45, CD34, and CD11b) (Figure 1C), confirming the identity and purity of the isolated hMSC population.

### 3.2. Characterization and Uptake of MSCs-Derived EVs

**Figure 2:**
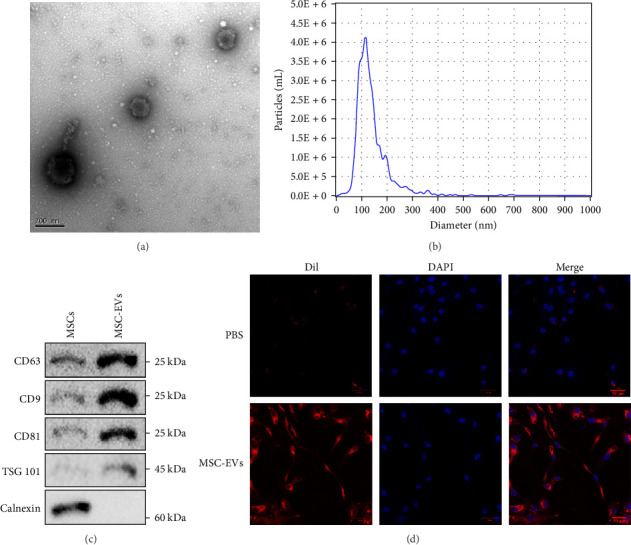
Characterization and uptake of MSCs-derived EVs. (A) Representative image of MSCs-EVs morphology by TEM (scale bar: 200 μm). (B) Particle size distribution of MSCs-EVs was measured by NTA. (C) Western blot analysis for EVs markers CD9, CD63, CD81, TSG101, and calnexin. (D) Uptake of dil-labeled MSCs-EVs by NPCs (scale bar: 50 μm). EVs, extracellular vesicles; MSCs, mesenchymal stem cells; TEM, transmission electron microscopy.

EVs were isolated from MSCs cultures, and their morphology was characterized using TEM (Figure 2A). NTA revealed a skewed particle size distribution, with a peak around 100 nm (Figure 2B), consistent with the expected size range for exosomes. Western blot analysis detected the expression of exosomal marker proteins, including CD63, CD9, CD81, and TSG101, in the isolated MSCs-EVs, while the endoplasmic reticulum protein calnexin was absent (Figure 2C), confirming the purity of the EVs preparation. To investigate the potential uptake of MSC-EVs by NPCs, dil-labeled MSCs-EVs were incubated with NPCs, and confocal microscopy revealed the co-localization of the labeled EVs with NPCs (Figure 2D), indicating successful internalization of the EVs by the recipient cells. These results demonstrate the successful isolation and characterization of MSCs-derived EVs and their ability to be taken up by NPCs, laying the foundation for further studies on the potential therapeutic effects of these EVs in IDD.

### 3.3. Analysis of Endoplasmic Reticulum Stress and Apoptosis in Degenerated Nucleus Pulposus Tissues

Degenerated NP tissues were examined by histological staining techniques. H&E, alcian blue, and masson's trichrome staining revealed varying degrees of degeneration, ranging from grade II to grade IV (Figure S1A). Normal and degenerated NP tissues across different grades of degeneration (I to V) were further analyzed by western blotting to assess the expression of ER stress-related proteins. Compared to normal NP tissues (NC), degenerated NP tissues (IDD model) exhibited higher expression levels of CHOP, GRP78, and phosphorylated IRE1*α*, while total IRE1*α* levels remained similar across all samples (Figure S1B). Western blot analysis of apoptosis-related proteins demonstrated lower levels of the antiapoptotic protein Bcl-2 and higher levels of the proapoptotic proteins Bax and cleaved caspase-3 in IDD relative to NC. (Figure S1C). Consistent with these findings, confocal microscopy imaging and quantification of relative fluorescence intensity revealed elevated levels of cleaved caspase-3 expression in grade IV degenerated NP tissues compared to grade II tissues (Figure S1D). Further western blot analysis confirmed the increased expression of ER stress-related proteins (Figure S1E) and apoptotic proteins (Figure S1F) in grade IV NP tissues relative to grade II tissues. These results collectively demonstrate the presence of heightened ER stress and apoptosis in degenerated NP tissues, with more advanced stages of degeneration exhibiting higher levels of these pathological processes.

### 3.4. MSCs-EVs Attenuated AGE-Induced ER-Stress, Apoptosis, and Inhibit ECM Degradation

**Figure 3:**
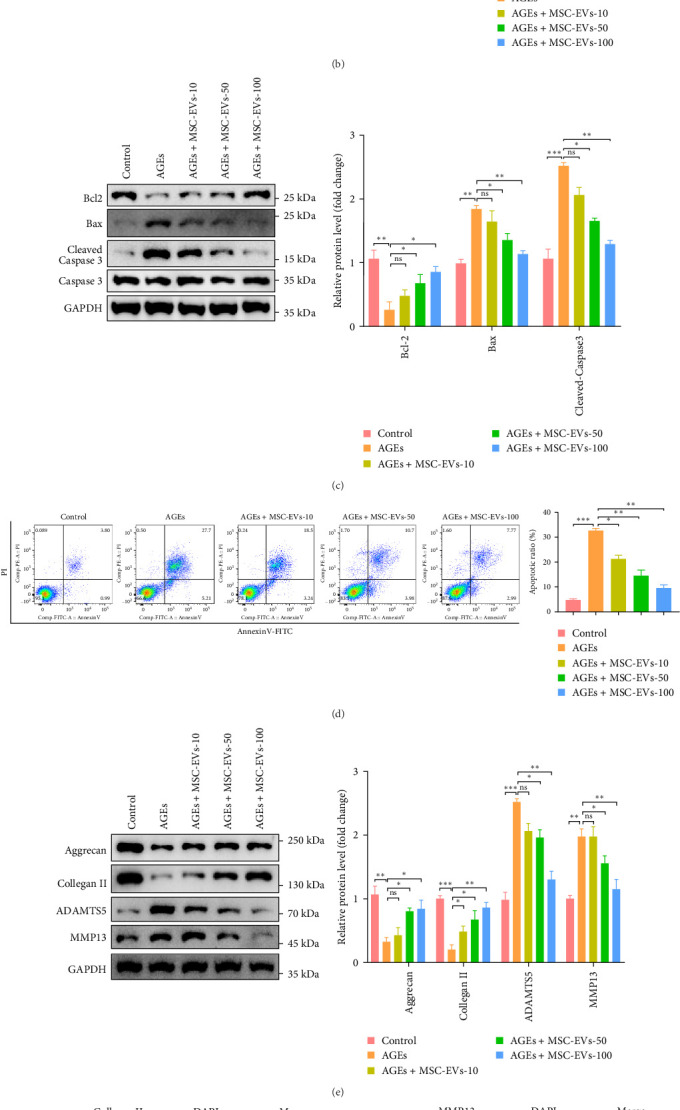
MSCs-EVs attenuated AGE-induced ER-stress, apoptosis, and inhibit ECM degradation. Human NPCs were treated with AGEs (200 μg/mL) for 24 h, but not the control group. 10, 50, or 100 μg/mL EVs was used, indicated by MSCs-EVs-10, 50, 100. (A). The protein levels of ER-stress were measured by Western blot analysis, and the relative quantitative data were calculated. GAPDH was used as an internal control. (B). Cell proliferation was determined using EdU staining combined with DAPI staining for the nuclei, and the positive cell ratio was calculatated (scale bar: 50 μm). (C). Western blotting and quantitative analysis of apoptosis-related protein levels were performed. GAPDH was used as an internal control. (D). Representative dot plot images by flow cytometry analysis after labeling with Annexin V-FITC and staining with PI, showing both early and late apoptosis cells; fluorescent intensities were quantified. (E). The protein expression of aggrecan, collagen II, MMP13, and ADAMTS5 were detected by Western blotting in NPCs; the relative quantitative data were calculated. (F-G). Levels of col II and MMP13 were evaluated by immunofluorescence (scale bar: 50 μm). *N* = 3 for all experiments. All data are presented as mean ± SD. *⁣*^*∗*^*p* < 0.05, *⁣*^*∗∗*^*p* < 0.01, *⁣*^*∗∗∗*^*p* < 0.001 compared with the indicated groups. AGEs, advanced glycation end products; ECM, extracellular matrix; ER, endoplasmic reticulum; EVs, extracellular vesicles; MSCs, mesenchymal stem cells; NPCs, nucleus pulposus cells.

To investigate whether MSCs-EVs can mitigate AGE-induced ER stress in NPCs, NPCs were pretreated with AGEs for 24 h and subsequently co-incubated with varying concentrations (10, 50, or 100 μg/mL) of MSCs-EVs in the presence of AGEs. Western blot analysis of ER stress-related proteins revealed that AGE treatment induced the expression of GRP78, CHOP, and phosphorylated IRE1*α*, while co-incubation with MSCs-EVs dose-dependently reduced the levels of these proteins. Total IRE1*α* expression remained similar across all samples (Figure 3A). EdU staining and immunofluorescence imaging demonstrated that AGE treatment reduced NPC proliferation, an effect that was attenuated by MSCs-EV co-incubation in a dose-dependent manner (Figure 3B). Examination of apoptosis-related proteins showed that AGE treatment decreased the antiapoptotic protein Bcl-2 and increased the pro-apoptotic proteins Bax and cleaved caspase-3, while co-incubation with MSCs-EVs dose-dependently reversed these effects (Figure 3C). Consistent with these findings, flow cytometric analysis using Annexin V and propidium iodide staining revealed a reduction in the percentage of apoptotic cells upon MSCs-EV treatment (Figure 3D). Furthermore, AGEs decreased ECM proteins (aggrecan, collagen II) but increased matrix-degrading enzymes (MMP13, ADAMTS5), which were reversed by MSC-EVs in a dose-dependent manner, confirmed by Western blot and immunofluorescence imaging (Figure 3). In summary, these results demonstrate that MSCs-derived EVs exert protective effects against AGE-induced ER stress, apoptosis, and ECM degradation in NPCs, with higher EV concentrations conferring greater therapeutic benefits. These findings suggest a potential role for MSCs-EVs in mitigating the detrimental effects of AGE accumulation and associated pathological processes in IDD.

### 3.5. Expression Profiling of miR-1275 in IDD

**Figure 4:**
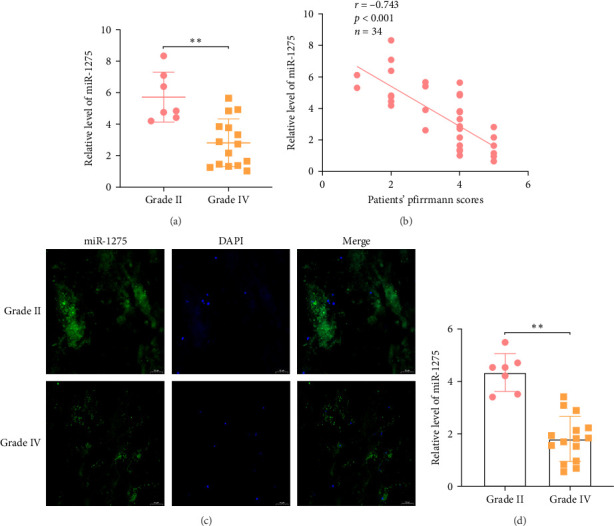
miR-1275 is downregulated in IDD. (A). Hsa-miR-1275 expression measured by qRT-PCR in human NP tissues. (B). Correlation between miR-1275 expression and Pfirrmann grade score of IDD was determined (*n* = 34, r = −0.743, *p* < 0.001). (C). FISH analysis for the expression of miR-1275 in NP tissues from patients with IDD (scale bar: 50 μm). (D). The downregulation of miR-1275 in cultured NPCs fromgrade IV tissues but not those from grade II tissues. All data are presented as mean ± SD. *⁣*^*∗∗*^*p* < 0.01, compared with the indicated groups. IDD, intervertebral disc degeneration; NPCs, nucleus pulposus cells.

The dysregulation of miRNAs expression levels can provide insights into the molecular mechanisms underlying IDD. Previous studies have implicated miR-1275 in regulating various cellular processes relevant to IDD pathogenesis, including the proliferation and chondrogenic differentiation of synovial fluid-derived mesenchymal stem cells. Notably, miR-1275 has been shown to modulate the expression of matrix metalloproteinase 13 (MMP13), a key catabolic enzyme involved in the degradation of extracellular matrix components within the disc [19]. Moreover, investigations in various cancers have demonstrated the ability of miR-1275 to promote cell proliferation [20–22], a process that is critical for the maintenance and regeneration of the NPC population during IDD progression. Considering the pivotal roles of NPC proliferation and chondrogenic matrix synthesis in preserving the biomechanical functions of the NP, elucidating the mechanistic involvement of miR-1275 in these processes could provide valuable insights into the pathogenesis of IDD and potentially identify novel therapeutic targets. qRT-PCR analysis further confirmed the decreased expression of miR-1275 in human NP tissues exhibiting grade IV degeneration relative to those with grade II degeneration (Figure 4A). Moreover, a significant negative correlation was observed between miR-1275 levels and the Pfirrmann scores of IDD across 34 patient samples (Figure 4 = −0.743, *p*  < 0.001), indicating an inverse relationship between miR-1275 expression and the severity of disc degeneration. FISH analysis and immunofluorescence imaging demonstrated the downregulation of miR-1275 in NP tissues obtained from patients with IDD compared to non-degenerated controls (Figure 4C). Consistent with these findings, cultured NP cells isolated from grade IV degenerated tissues exhibited lower miR-1275 expression levels compared to those derived from grade II tissues (Figure 4D).These comprehensive analyses across multiple platforms and sample types consistently revealed the downregulation of miR-1275 in IDD, suggesting a potential regulatory role for this miRNA in the pathogenesis of disc degeneration. Further investigations into the molecular targets and functional implications of miR-1275 dysregulation may provide valuable insights into the underlying mechanisms driving IDD and identify novel therapeutic strategies.

### 3.6. miR-1275 Shuttled by MSCs-EVs Promoted Matrix Synthesis and Proliferation of NPCs

**Figure 5:**
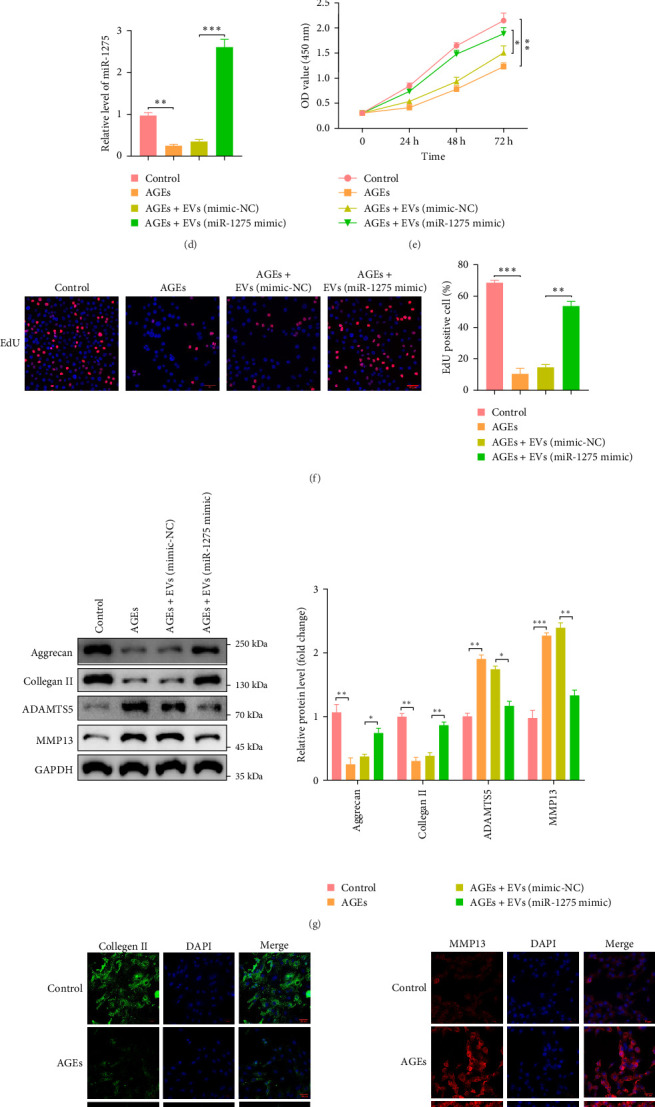
miR-1275 shuttled by MSCs-EVs promoted matrix synthesis and proliferation of NPCs. (A) Uptake of MSCs-EVs with Cy3-labeled miR-1275 by NPCs (scale bar: 50 μm). (B) miR-1275 expression was determined in NPCs by qRT-PCR after treatment with different concentrations ofMSCs-EVs. (C) miR-1275 expression in EVs determined by qRT-PCR from miR-1275 mimic- or its normal control-transfected MSCs. (D) miR-1275 level in AGEs-induced NPCs which were co-cultured with EVs derived from miR-1275-transfected MSCs. (E) CCK-8 assay for the cell proliferation of AGEs-induced and EVs-treated NPCs. (F) Cell proliferation was determined using EdU staining (red) combined with DAPI staining for the nuclei (blue), scale bar: 50 μm. (G) Western blot analysis for the protein levels of col II, aggrecan, MMP13, and ADAMTS-5 in AGEs-induced NPCs which were co-cultured with EVs derived from miR-1275 mimic or its normal control (NC) transfected MSCs; the relative quantitative data were presented in the bar chart. (H-I) immunofluorescence staining for the expressions of col II (green) and MMP13 (red) in AGEs-induced NPCs after co-culture with EVs or miR-1275-EVs(scale bar: 50 μm). All data are presented as mean ± SD. *⁣*^*∗*^*p* < 0.05, *⁣*^*∗∗*^*p* < 0.01, *⁣*^*∗∗∗*^*p* < 0.001 compared with the indicated groups. AGEs, advanced glycation end products; EVs, extracellular vesicles; MSCs, mesenchymal stem cells; NPCs, nucleus pulposus cells.

Immunofluorescence imaging revealed the presence of Cy3-labeled miR-1275 (red) within MSCs-EVs, which co-localized with the nuclei (DAPI staining, blue) of NPCs, suggesting the internalization of miR-1275-containing MSCs-EVs by NPCs (Figure 5A). qRT-PCR analysis demonstrated that AGE treatment reduced miR-1275 expression levels in NPCs, an effect that was reversed in a dose-dependent manner upon co-incubation with varying concentrations of MSCs-EVs (Figure 5B). *In vitro* experiments confirmed that transfection of MSCs with miR-1275 mimics significantly increased the levels of miR-1275 within the derived EVs (Figure 5C). While AGE treatment decreased miR-1275 levels in NPCs, co-incubation with miR-1275 mimic EVs counteracted this effect (Figure 5D). Functional assays revealed that AGE exposure reduced NPC proliferation in a time-dependent manner up to 72 h, an effect that was rescued by co-incubation with miR-1275-enriched MSCs-EVs but not by control EVs (Figure 5E, F). Consistent with these findings, western blot and immunofluorescence analyses showed that AGE treatment downregulated the expression of the extracellular matrix proteins aggrecan and collagen II while upregulating the matrix-degrading enzymes MMP13 and ADAMTS5 in NPCs. Remarkably, miR-1275 EVs reversed these effects (Figure 5). Collectively, these data demonstrate that miR-1275-enriched MSCs-EVs are internalized by NPCs, mitigating AGE-induced ER stress, apoptosis, and matrix degradation. These findings suggest a regulatory role for miR-1275 in modulating NPCs function and indicate its potential involvement in the therapeutic effects of MSCs-EVs in the context of IDD.

### 3.7. EVs-MiR-1275 Alleviates ER-Stress by Promoting ER-Phagy

**Figure 6:**
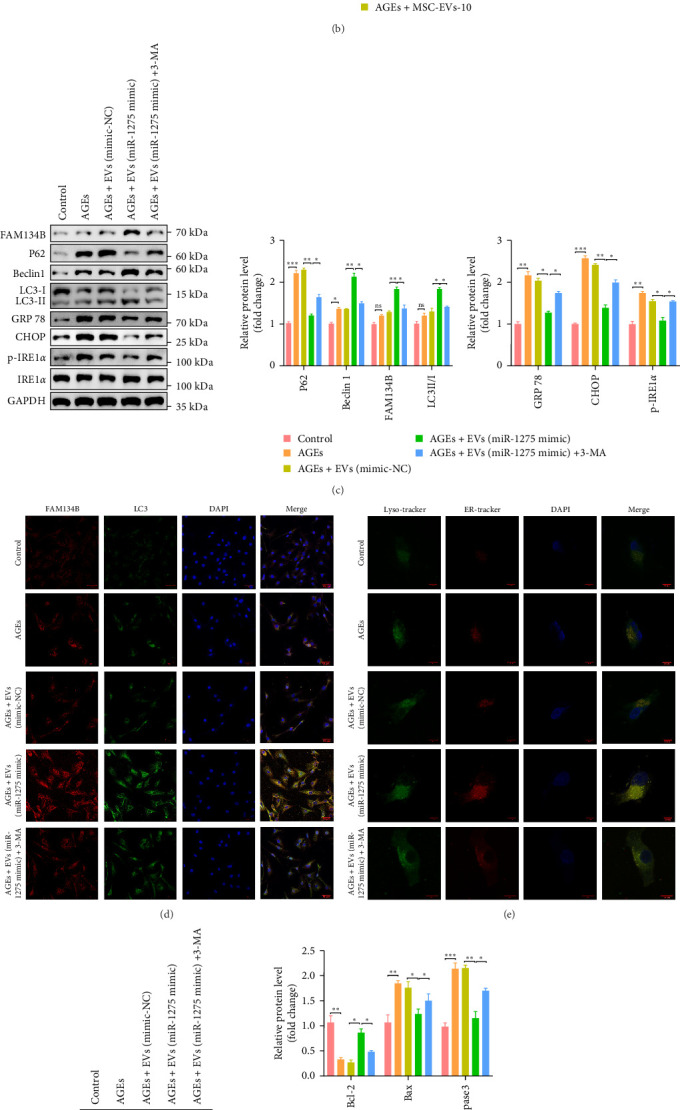
EVs-miR-1275 alleviates ER-stress by promoting ER-phagy. (A) Expression of FAM134B, p62, Beclin1, LC3II/I proteins in normal and degenerative NP tissues were analyzed by Western blotting. (B) The protein expressions of FAM134B, p62, Beclin1, LC3II/I in NPCs treated with different concentrations of MSCs-EVs were visualized by Western blotting; the relative quantitative data were calculated in the bar chart. (C) AGEs- and 3-MA (10 mM)-induced NPCs were treated with EVs derived from miR-1275-transfected MSCs to detect the expression of ER-phagy and ER-stress proteins by Western blotting; the relative quantitative data were calculated in the bar chart. (D) Representative fluorescent images of staining for FAM134B (red) and LC3 (green), and cell nuclei were stained with DAPI (blue),scale bar: 50 μm. (E) ER and lysosome colocalization was analyzed by ER-tracker (green) and Lyso-tracker (red) staining (scale bar: 10 μm). (F) Representative Western blots and quantitative analysis of apoptosis-related protein levels. GAPDH was used as an internal control. (G) Apoptosis of NPCs detected by flow cytometry. All data are presented as mean ± SD. *⁣*^*∗*^*p* < 0.05, *⁣*^*∗∗*^*p* < 0.01, *⁣*^*∗∗∗*^*p* < 0.001 compared with the indicated groups. AGEs, advanced glycation end products; ER, endoplasmic reticulum; EVs, extracellular vesicles; MSCs, mesenchymal stem cells; NPCs, nucleus pulposus cells.

Western blot analysis revealed dysregulated expression of ER-phagy markers in degenerated NPCs compared to normal cells. Specifically, the protein levels of FAM134B, beclin1, and LC3II/I were downregulated, while p62 expression was evidently upregulated in degenerated NPCs (Figure 6A). However, co-incubation of NPCs with AGEs and increasing concentrations of MSCs-EVs resulted in a dose-dependent increase in FAM134B, beclin1, and LC3II/I levels. Conversely, the AGE-induced upregulation of p62 was dose-dependently attenuated by MSCs-EV supplementation (Figure 6B), suggesting that FAM134B, beclin1, and LC3II/I mediate MSCs-EV-induced ER-phagy. Further mechanistic studies using miR-1275-enriched EVs (EVs-miR-1275) corroborated these findings. Incubation of NPCs with AGEs and EVs-miR-1275 reduced p62 levels while increasing beclin1, FAM134B, and LC3II/I expression compared to AGE treatment alone or co-incubation with control EVs (EVs-NC). However, co-treatment with the autophagy inhibitor 3-MA diminished the upregulation of beclin1, FAM134B, and LC3II/I in NPCs exposed to AGEs and EVs-miR-1275 (Figure 6C). Similarly, AGE treatment induced ER stress markers (GRP78, CHOP, and phosphorylated IRE1*α*), which were downregulated upon co-incubation with EVs-miR-1275. Notably, 3-MA co-treatment restored the AGE-induced upregulation of ER stress markers in NPCs treated with AGEs and EVs-miR-1275 (Figure 6C). Confocal microscopy confirmed the upregulation of FAM134B (red) and LC3II/I (green) in NPCs incubated with AGEs and EVs-miR-1275, an effect that was abrogated by 3-MA (Figure 6D). Moreover, co-localization of ER (green) and lysosome (red) markers was most pronounced in NPCs treated with AGEs and EVs-miR-1275 compared to other treatment conditions (Figure 6E). Evaluation of apoptotic markers revealed that EVs-miR-1275 reduced the levels of proapoptotic proteins Bax and cleaved caspase-3 while increasing the antiapoptotic protein Bcl-2 in AGE-treated NPCs. Conversely, 3-MA co-treatment increased Bax and cleaved caspase-3 levels while decreasing Bcl-2 expression in NPCs exposed to AGEs and EVs-miR-1275 (Figure 6F). Flow cytometric analysis of Annexin V and propidium iodide staining corroborated these findings (Figure 6G). Collectively, these data demonstrate that MSCs-EVs, particularly those enriched in miR-1275, induce ER-phagy in NPCs, as evidenced by the modulation of key autophagy markers. This ER-phagy induction appears to mediate the protective effects of MSCs-EVs against AGE-induced ER stress and apoptosis in NPCs, highlighting a potential therapeutic mechanism underlying the beneficial actions of MSCs-EVs in the context of IDD.

### 3.8. Bioinformatic Analysis Identifies AXIN2 as a Target of miR-1275

**Figure 7:**
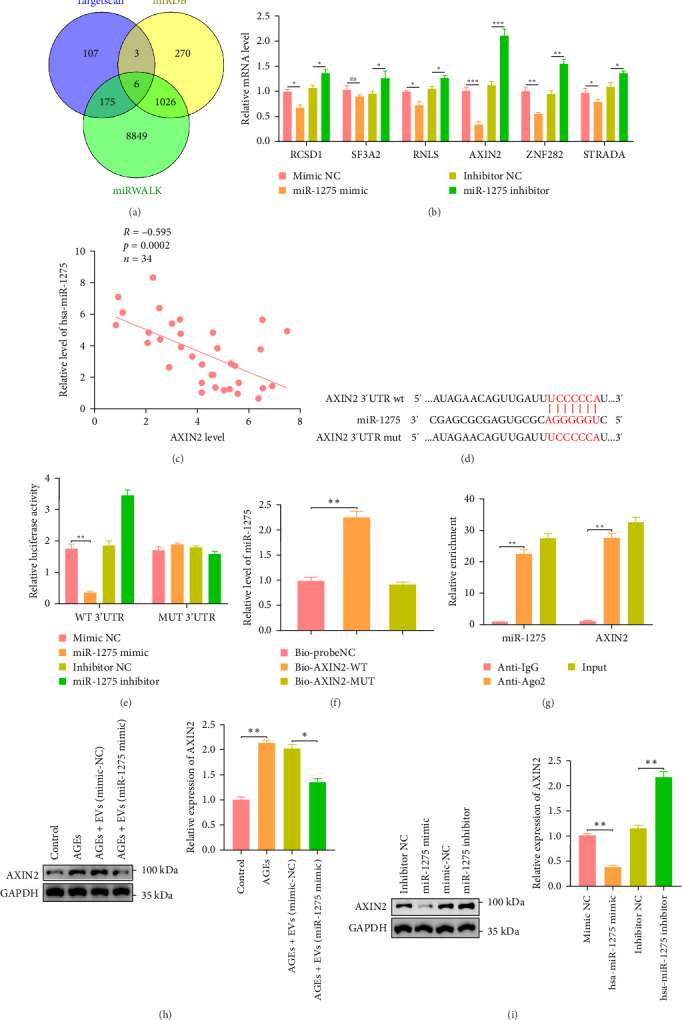
Bioinformatic analysis identifies AXIN2 as a target of miR-1275. (A) Using TargetScan, miRDB, and miRWALK databases screening of target genes for miR-1275 and the middle part represents the intersecting genes from them. (B) RCSD1, SF3A2, RNLS, AXIN2, ZNF282, and STRADA mRNA levels in NPCs in response to miR-1275 mimic or miR-1275 inhibitor determined by qRT-PCR. (C) Correlation of hsa-miR-1275 expression level with AXIN2 in IDD (*n* = 34, *r* = −0.595, *p*=0.0002). (D) Identification of a putative miR-1275 binding site in the 3′-UTR regions of AXIN2 by Targetscan. (E) The relationship between miR-1275 and AXIN2 examined by dual-luciferase reporter gene assay (*⁣*_*∗*_*p* < 0.05, vs., mimic NC). (F) The relationship between miR-1275 and AXIN2 studied by RNA pull-down assay (*⁣*_*∗*_*p* < 0.05, vs., Bioprobe NC or Bio-AXIN2-MUT). (G) The interaction between miR-1275 and AXIN2 was examined by RIP assay (*⁣*_*∗*_*p* < 0.05,vs., mimic NC). (H) AGEs-induced NPCs were treated with EVs derived from miR-1275-transfected MSCs to detect the expression of AXIN2 by Western blot analysis. (I) AXIN2 protein levels in NPCs in response to hsa-miR-1275 mimic or miR-1275 inhibitor determined by Western blot analysis; results were quantified. All data are represented as mean ± SD. *⁣*^*∗*^*p* < 0.05, *⁣*^*∗∗*^*p* < 0.01, *⁣*^*∗∗∗*^*p* < 0.001, compared with the indicated groups. AGEs, advanced glycation end products; IDD, intervertebral disc degeneration; MSCs, mesenchymal stem cells; NPCs, nucleus pulposus cells.

To explore the target genes of miR-1275, predictive scores from TargetScan, miRDB, and miRWalk were plotted, revealing six common intersections (RCSD1, SF3A2, RNLS, AXIN2, ZNF282, and STRADA) (Figure 7A). qRT-PCR analysis showed that under conditions of miR-1275 overexpression or inhibition, the mRNA levels of the identified target AXIN2 exhibited the most significant changes in NPCs (Figure 7B), suggesting that AXIN2 is a target gene of miR-1275. Further analysis revealed a negative correlation (*r* = −0.595, *p*=0.0002) between miR-1275 and AXIN2 levels in 34 IDD patient samples (Figure 7C). TargetScan identified a putative miR-1275 binding site within the 3′UTR of wild-type AXIN2, which was absent in a mutated AXIN2 construct (Figure 7D). Dual-luciferase reporter assays in NPCs transfected with miR-1275 and the wild-type AXIN2 3′UTR demonstrated reduced AXIN2 expression, an effect that was reversed by miR-1275 inhibition. Co-transfection with the mutated AXIN2 3′UTR had no impact on AXIN2 levels (Figure 7E). A pull-down assay using biotin-labeled wild-type AXIN2 confirmed binding to miR-1275, while no interaction was observed with the mutated AXIN2 construct (Figure 7F). Presence of miR-1275 and AXIN2 was confirmed in immunoprecipitates for Ago2 (a protein that binds to miRNA). Levels of miR-1275 and AXIN2 in immunoprecipitates were compared to the input levels, indicating that all mi-1275 and AXIN2 molecules were bound to Ago2. No presence of miR-1275 and AXIN2 was detected in immunoprecipitates for IgG (Figure 7G). Western blot analyses showed reduced AXIN2 protein levels in NPCs treated with AGEs and EVs-miR-1275 compared to AGE treatment alone (Figure 7H). Furthermore, miR-1275 overexpression in NPCs decreased AXIN2 protein levels, while miR-1275 inhibition led to AXIN2 upregulation (Figure 7I). Collectively, these data validate AXIN2 as a direct target of miR-1275 in NPCs.

### 3.9. AXIN2 Negatively Regulates miR-1275-Mediated Effects on ER-Phagy, ER Stress, and Apoptosis

**Figure 8:**
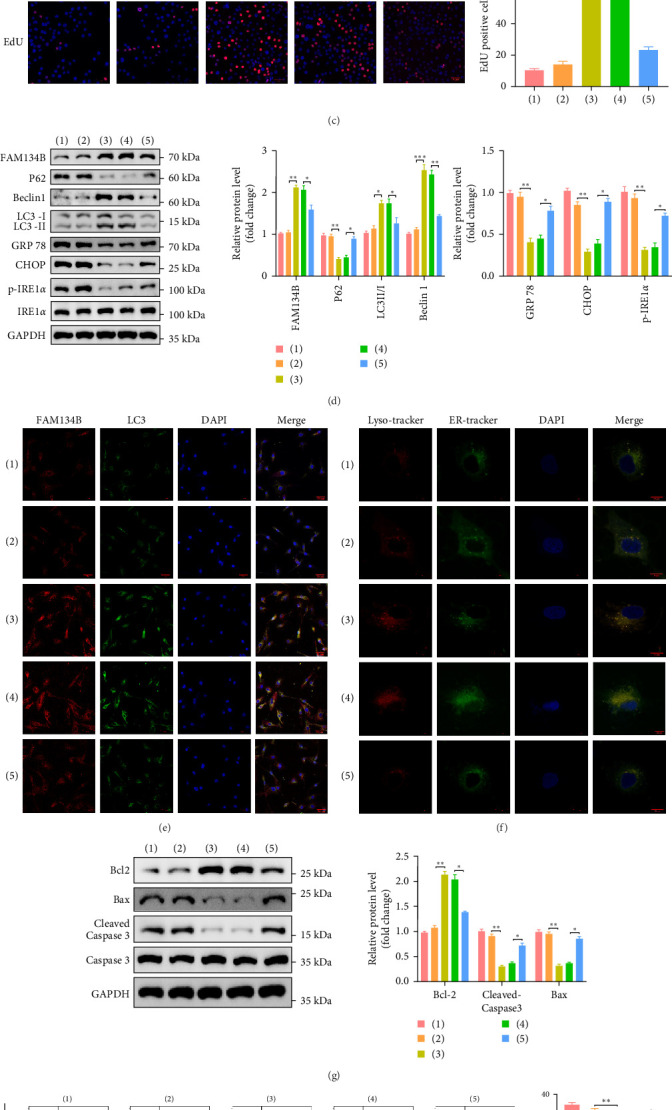
miR-1275 targeting AXIN2 suppresses apoptosis by promoting ER-phagy in NPCs. (A) AGEs-induced NPCs were transfected with miR-1275 mimic or in combination with AXIN2. miR-1275 expression detected by qRT-PCR in NPCs. (B) AXIN2 mRNA expression detected by qRT-PCR in NPCs. (C) Cell proliferation was determined using EdU staining (red) combined with DAPI staining for the nuclei (blue), and the positive cell ratio was calculated (scale bar: 50 μm). (D) Detection of the expression of ER-phagy and ER-stress proteins by Western blotting and the results were quantified in the bar chart. (E) Representative fluorescent images of staining for FAM134B (red) and LC3 (green), and cell nuclei were stained with DAPI (blue), scale bar: 50 μm. (F) ER and lysosome colocalization was studied by ER-tracker (green) and Lyso-tracker staining (red), scale bar: 10 μm. (G) Western blot analysis of the levels of apoptosis-related protein and results were quantified in the bar chart. GAPDH was used as an internal control. (H) Apoptosis of NPCs was detected by flow cytometry. All data are presented as mean ± SD. *⁣*^*∗*^*p* < 0.05, *⁣*^*∗∗*^*p* < 0.01, *⁣*^*∗∗∗*^*p* < 0.001 compared with the indicated groups. AGE, advanced glycation end products; ER, endoplasmic reticulum; NPCs, nucleus pulposus cells.

To delineate the functional interplay between miR-1275 and its target AXIN2, rescue experiments were performed in an AGE-induced NPC model of IDD. NPCs were divided into five experimental groups: AGE treatment alone, AGE + mimic NC, AGE + miR-1275 mimic, AGE + miR-1275 mimic + AXIN2 overexpression. Successful modulation of miR-1275 and AXIN2 levels was confirmed by qRT-PCR, which showed increased miR-1275 expression upon mimic transfection that was attenuated by AXIN2 overexpression (Figure 8A). Conversely, AXIN2 mRNA levels were downregulated by miR-1275 overexpression but restored in the AXIN2 rescue group (Figure 8B). Cell proliferation assays using EdU labeling revealed enhanced NPCs proliferation upon miR-1275 overexpression, an effect that was abrogated by concomitant AXIN2 overexpression (Figure 8C). Western blot analyses demonstrated upregulated protein levels of the ER-phagy markers FAM134B, beclin1, and LC3II/I in miR-1275 overexpressing NPCs. However, these inductions were diminished when AXIN2 was co-overexpressed (Figure 8D). Conversely, the autophagy substrate p62 exhibited the opposite expression pattern. Interestingly, the ER stress response proteins GRP78, CHOP, and phosphorylated IRE1*α* were downregulated by miR-1275 overexpression but upregulated upon AXIN2 rescue (Figure 8D). These findings were corroborated by immunofluorescence analyses of FAM134B and LC3 (Figure 8E). Furthermore, co-localization of ER and lysosome markers, indicative of productive ER-phagy flux, was prominent in miR-1275 overexpressing NPCs and mimic NC controls but attenuated in the AXIN2 rescue group (Figure 8F). Apoptosis assays revealed reduced levels of the proapoptotic proteins Bax and cleaved caspase-3 upon miR-1275 overexpression, which were upregulated by AXIN2 co-overexpression. Conversely, the antiapoptotic Bcl-2 protein exhibited an opposing expression pattern (Figure 8G). Flow cytometric analysis using Annexin V and propidium iodide staining further corroborated the effects on apoptosis modulation (Figure 8H). Collectively, these rescue experiments establish that AXIN2 overexpression can negate the inductive effects of miR-1275 on ER-phagy, ER stress alleviation, and antiapoptotic signaling in AGE-challenged NPCs. These findings underscore the functional importance of the miR-1275-AXIN2 regulatory axis in modulating cellular homeostasis and survival in the context of IDD.

## 4. Discussion

The findings in this study demonstrated that MSCs-derived EVs shuttling miR-1275 as cargo improved ER-phagy activity in NPCs, thereby inhibiting ER-stress and reducing the levels of apoptosis. During ER-stress, unfolded/misfolded proteins accumulate in the ER lumen which in the long term may lead to degenerative processes in the cell [23, 24]. The process of ER-phagy can be an attempt to restore ER functions and homeostasis of the ER in general during ER-stress [25]. Indeed, previous studies have demonstrated that ER-phagy plays a important role in AGEs-induced apoptosis and senescence in NPCs and that targeting of ER-phagy could therefore be a potential target for treatment of IDD [26]. This is in line with other studies that suggest that ER-phagy is a pivotal intracellular regulatory mechanism of homeostasis and thereby regulates cell function and fate under different stress conditions [27, 28]. Our data corroborate these previous findings and provide mechanistic insights into the regulatory role of miR-1275 in modulating ER-phagy and ER stress in NPCs. Notably, we observed that miR-1275 overexpression enhanced the expression of key ER-phagy markers, such as FAM134B, beclin1, and LC3-II, while concomitantly alleviating ER stress, as evidenced by the downregulation of GRP78, CHOP, and phospho-IRE1*α*. Importantly, these effects were abrogated upon rescue with AXIN2 overexpression, substantiating the functional interplay between miR-1275 and its target AXIN2 in governing ER homeostasis and survival signaling in NPCs.

In search for miRNAs that had an effect on AGE-induced apotosis and ER-stress in NPCs as a model for IDD, miR-1275 was identified. miR-1275 is a confirmed tumor-respressing miRNA and plays an important role in several diseases, including obesity [29] and cancer [30, 31]. Previous studies have revealed diverse regulatory roles of miR-1275 across different pathological contexts. In colorectal cancer, miR-1275 was found to be enriched in ECM–receptor interactions, suggesting its involvement in extracellular matrix regulation [32]. In prostate cancer, exosomal miR-1275 secreted by cancer cells modulates osteoblast proliferation and activity through targeting the SIRT2/RUNX2 cascade [33]. Additionally, research in breast cancer has demonstrated that circCDYL promotes autophagy through the miR-1275-ATG7/ULK1 axis [34]. These findings collectively highlight the multifaceted functions of miR-1275 in regulating cell proliferation, ECM, and autophagy across different pathological conditions.

In our study, we found that miR-1275 mediates its effect in NPCs by its target protein AXIN2. A previous study also showed the miR-1275/AXIN2 axis in bladder cancer tissues. miR-1275 negatively regulated AXIN2 levels in these cells, and AXIN2 in its turn was a negative regulator of the Wnt/*β*-catenin pathway [35]. Interestingly, our findings unveil a novel layer of regulation, wherein miR-1275 modulates ER homeostasis and cellular survival in NPCs, at least in part, by targeting AXIN2. This expands the known functions of the miR-1275/AXIN2 axis beyond its established roles in cancer and highlights its potential therapeutic implications in degenerative disorders such as IDD.

A study by Smolders et al. has reported on the presence and role of AXIN2 in NP tissues. They observed that AXIN2 gene expression was significantly lower in chondrocyte-like cells (CLCs) compared to notochordal cells within the NP. The transition from NCs to CLCs is a hallmark of early IDD [36]. Consistently, AXIN2 expression was significantly downregulated in degenerated NP tissues. As a negative feedback regulator of the Wnt/*β*-catenin signaling pathway, AXIN2 serves as a reliable readout of pathway activity and participates in a negative feedback loop to limit the duration or intensity of Wnt signaling [37]. In agreement with the previous findings, early IDD degeneration involves downregulation of canonical Wnt signaling, suggesting that this pathway [38], regulated by AXIN2, is essential for the physiology and preservation of NPCs. Our results provide a potential mechanistic explanation for these previous observations. However, further investigations are warranted to delineate the intricate interplay between miR-1275, AXIN2, and Wnt signaling in the context of IDD pathogenesis.

One limitation of the current study design lies in the absence of *in vivo* animal experiments to corroborate the mechanistic findings pertaining to miR-1275. Our work thus far has primarily focused on *in vitro* cell culture models to delineate the molecular mechanisms underlying the development of IDD. While *in vitro* studies offer a controlled environment conducive to precise molecular manipulations, they inevitably fail to recapitulate the intricate complexities of living organisms with sophisticated biological systems and multiple interacting components. Specifically, the intervertebral disc microenvironment involves complex interactions between different cell types, mechanical forces, and various biochemical factors that cannot be fully replicated in cell culture systems. Additionally, the long-term effects of miR-1275 delivery via MSC-derived EVs, including potential off-target effects and systemic responses, can only be thoroughly evaluated through *in vivo* studies. Future studies should focus on developing advanced delivery systems, such as nanomaterials or hydrogels incorporating EVs, to achieve more efficient and controlled delivery of miR-1275 for IDD treatment. These biomaterial-based approaches could potentially enhance the therapeutic efficacy and provide better spatial and temporal control of miR-1275 delivery in the complex disc microenvironment. Moreover, compared to direct MSC transplantation, these delivery systems might reduce potential immunological responses while maintaining the therapeutic benefits, thereby offering a safer and more practical approach for clinical translation.

## 5. Conclusions

Altogether, our *in vitro* studies have identified a molecular mechanism, whereby miR-1275, delivered by MSCs-derived EVs, directly targets AXIN2 in NP cells. In our cellular model, we demonstrated that miR-1275 modulates key cellular processes by promoting ER-phagy, regulating NPC survival, and influencing ECM homeostasis through the regulation of Coll II, aggrecan, MMP13, and ADAMTS5 expression. These findings provide insights into the molecular pathways involved in IDD and suggest a potential role for miR-1275 in maintaining NPC homeostasis through the regulation of catabolic and anabolic factors. While further *in vivo* studies are needed, our cellular findings establish a foundation for understanding the molecular mechanisms of miR-1275 in NP cells and may inform future therapeutic strategies for IDD.

## Figures and Tables

**Table 1:** Primer sequences for qRT-PCR.

Genes	Forward primer (5′-3′)	Reverse primer (5′-3′)
miR-1275	GCGCGGTGGGGGAGAG	AGTGCAGGGTCCGAGGTATT
RCSD1	TGGGACCTGAACATGACAGC	GACATCTCCGCCCTTCACTC
SF3A2	CGACATCAACAAGGACCCGT	GTGCTTCTTCCCCTGCGTAT
RNLS	ATTGATGTCCCTTGGGCTGG	CGAGGGAAGGCCCAATTTCT
AXIN2	GACCCCACCGTGTCACCAT	GGAACAGGTAAGCACCGTCT
ZNF282	CTCAGCCACCGTCTGGAAAT	GCGTCCATTCAGACCAGACA
STRADA	GAGTGTCAAAGCCAGCCACA	GACTTGGCATCATAACCCTGCT

## Data Availability

All data supporting the results have been displayed in the paper itself or uploaded as Supporting Information for Online Publication.

## Supporting Information

Supporting Information Figure S1: provides additional image analysis supporting the main findings of the study.
