# Supplementary material for: miR-1275 Delivered via Mesenchymal Stem Cell-Derived Extracellular Vesicles Regulates ER-Phagy Through AXIN2 in Nucleus Pulposus Cells
Source: Stem Cells Int. 2025 May 29;2025:5091529. doi: 10.1155/sci/5091529 (PMC12140824; doi:10.1155/sci/5091529)
Supplement: Supporting Information — Figure S1: provides additional image analysis supporting the main findings of the study. [file 5091529.f1.docx]

**miR-1275 Delivered via Mesenchymal Stem Cell-Derived Extracellular Vesicles Regulates ER-Phagy through AXIN2 in Nucleus Pulposus Cells**

**
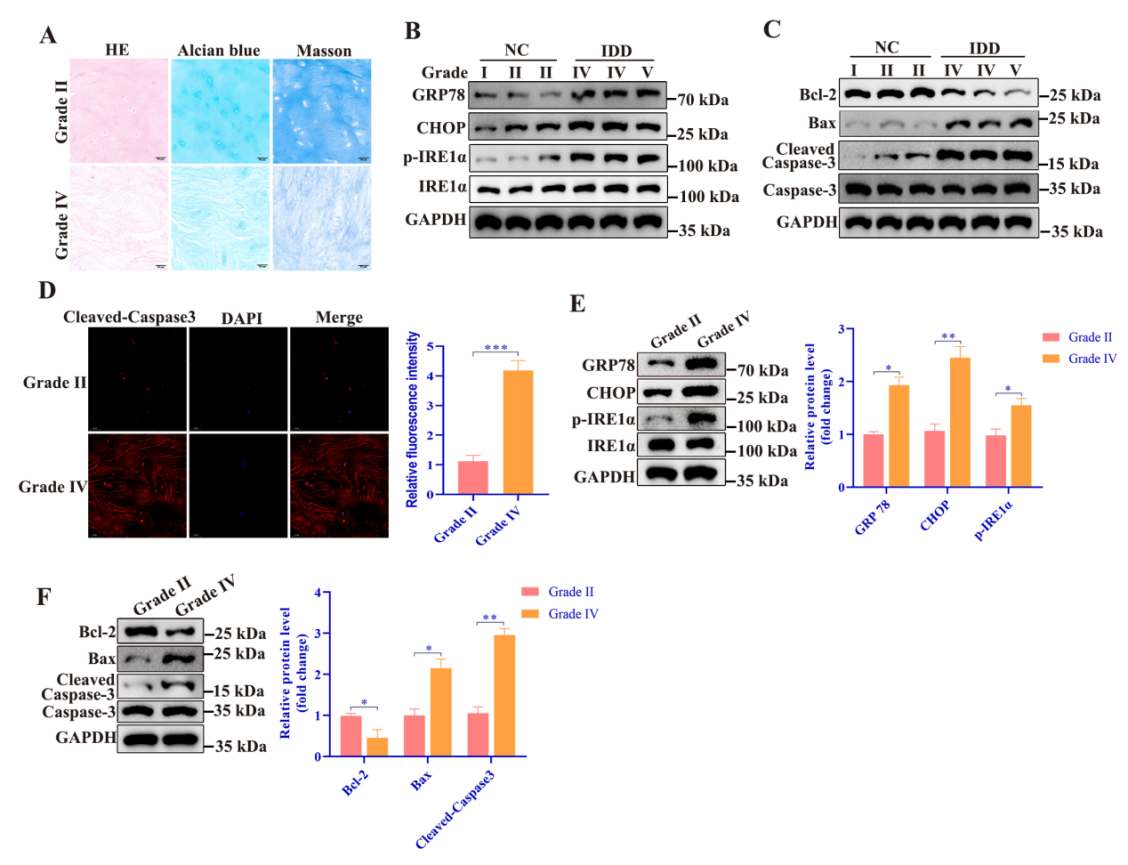
**

**Supplementary figure 1. Activation of ER-stress in IDD.** A. Representative histological images of grade II and grade IV NP tissues in H&E, Alcian blue and Masson staining (scale bar: 50 μm). B. Western blot analysis of the expression levels of ER-stress-related proteins. C. Western blot analysis of the expression levels of apoptosis-related proteins. D. Representative images of Cleaved Caspase-3 expression by immunofluorescence analysis; the relative fluorescence intensity was calculated in grade II and grade IV NP tissues (scale bar: 50 μm). E. Western blot analysis of the expression levels of ER-stress-related proteins (E) and apoptosis-related proteins (F) in grade II NPCs and grade IV NPCs and the relative quantitative data. All data are presented as mean ± S.D. ^*^*p*< 0.05, ^**^*p*< 0.01, ^***^*p*< 0.001 compared with the indicated groups.
